# Arginine Catabolic Mobile Element in Evolution and Pathogenicity of the Community-Associated Methicillin-Resistant *Staphylococcus aureus* Strain USA300

**DOI:** 10.3390/microorganisms8020275

**Published:** 2020-02-18

**Authors:** Kaiyu Wu, John Conly, Jo-Ann McClure, Habib A. Kurwa, Kunyan Zhang

**Affiliations:** 1 Department of Pathology & Laboratory Medicine, University of Calgary, Calgary, AL T2N 4N1, Canada; kaiyu.wu@ucalgary.ca (K.W.); john.conly@albertahealthservices.ca (J.C.); 2 Department of Microbiology, Immunology & Infectious Diseases, University of Calgary, Calgary, AL T2N 4N1, Canada; 3 Department of Medicine, University of Calgary, Calgary, AL T2N 4N1, Canada; Habib.Kurwa@albertahealthservices.ca; 4 The Calvin, Phoebe and Joan Snyder Institute for Chronic Diseases, University of Calgary, Calgary, AL T2N 4N1, Canada; 5 Centre for Antimicrobial Resistance, Alberta Health Services/Alberta Public Laboratories/University of Calgary, Calgary, AL T2N 4N1, Canada; joannmcc@shaw.ca

**Keywords:** ACME, *Staphylococcus aureus*, USA300, CA-MRSA, epidemiology, evolution, pathogenicity

## Abstract

USA300 is a predominant community-associated methicillin-resistant *Staphylococcus aureus* strain which carries an arginine catabolic mobile element (ACME). ACME contains potential virulence factors including an arginine deiminase (*arc*) pathway and an oligopeptide permease (*opp-3*) system, which are proposed to play a role in bacterial virulence and transmission. However, the role of ACME in evolution and pathogenicity of USA300 remains to be elucidated. ACME and *arcA* deletion mutants were created by allelic replacement from a USA300 clinical isolate. By comparing wild type and isogenic ACME deletion USA300 strains, ACME was shown not to contribute to bacterial survival on plastic surfaces, and mouse skin surfaces. ACME did not contribute to bacterial virulence in cell invasion and cytotoxicity assays, invertebrate killing assays and a mouse skin infection model. Wild-type ACME negative USA300 clinical isolates showed similar associations with invasive anatomic sites as ACME positive isolates. Our experiments also demonstrated that ACME can spontaneously excise from the bacterial chromosome to generate an ACME deletion strain at a low frequency. Our results do not support that the ACME element alone is a significant factor in the transmission and virulence of USA300 strain, and ACME may have been coincidently incorporated into the genome of USA300.

## 1. Introduction

*Staphylococcus aureus* is a Gram-positive, coagulase-positive coccus of approximately 1 µm in diameter forming grape-like clusters. It is a facultative anaerobe and can grow in the presence of 10% NaCl between 18–40 °C and in range of pH 4.0–9.8 with an optimum pH 6–7. Community-associated methicillin-resistant *S. aureus* (CA-MRSA) strain USA300, mostly associated with skin and soft tissue infections of people in the community setting, is geographically wide-spread, reported in all continents except Antarctica [1,2,3]. Diep et al. sequenced a USA300 clinical isolate and revealed that it carries a unique mobile genetic element, arginine catabolic mobile element (ACME) [4]. ACME contains *arc* and *opp-3* operons encoding the arginine deiminase pathway and the oligopeptide permease system, respectively [4]. A previous study has shown that the arginine deiminase pathway could enhance bacterial tolerance to acid in low pH (pH 4.0) by producing ammonia [5]. Arginine deiminase, a key enzyme in this catabolic pathway, is a virulence factor in *Streptococcus pyogenes*, which inhibits proliferation of human T-cells [6] and enhances bacterial invasion and survival at low pH intracellular environment [7]. Moreover, *Opp* operons are found in both Gram-positive and negative bacteria and their major function is to uptake peptides to be used as carbon and nitrogen sources. Many other functions have been indicated for these two components, including quorum sensing, chemotaxis, eukaryotic cell adhesion, binding of serum components, and expression of virulence determinants through peptide transport [8]. Recently, SpeG encoded by ACME was suggested to play a role in resistance to clearance by host [9]. Thus, ACME is hypothesized to enhance USA300 virulence and survival, supported by a study showing that ACME is associated with enhanced fitness of USA300 in a rabbit bacteremia model [10]. However, using a rodent model of necrotizing pneumonia and skin infection, Montgomery et al. demonstrated no difference in survival, bacterial burden and appearances of lesions among the wild type (WT), isogenic ACME deletion mutant strains and ACME negative (ACME^−^) USA300 clinical isolates, suggesting that ACME is not necessary for the virulence of USA300 in these models [11]. The contradictory results indicate that the precise role of ACME in CA-MRSA infection remains to be fully elucidated. Furthermore, the role of ACME in bacterial colonization and transmission, which is another significant characteristic of USA300 strains, has not been investigated to date.

In the present study, we created ACME and *arc*A deletion mutants of a USA300 clinical isolate obtained from a patient with necrotizing pneumonia from a local outbreak in 2004 [12]. Our results have demonstrated no difference between WT and the ACME and *arc*A deletion mutants in survival and virulence, suggesting that ACME may not be a key factor contributing to bacterial colonization/transmission and virulence of USA300.

## 2. Materials and Methods 

### 2.1. Bacterial Strains and Plasmids

*S. aureus* USA300-2406 is a clinical isolate from a patient with necrotizing pneumonia which occurred during an outbreak in Calgary in 2004. USA300-2406 has the same pulsed field gel electrophoresis (PFGE) pattern and the other molecular characteristics as USA300-0114, a typical PFGE subtype of USA300 isolates [4,12]. CMRSA-7 is a USA400 reference strain from the National Microbiology Laboratory, Canada, and M92 is a nasal colonizing strain (isolated from Calgary hospitals) that is not associated with infection. The plasmid pBT2 (a kind gift from Dr. Brückner at the University of Kaiserslautern, Germany [13]) and pSR2 (a kind gift from Dr. Ito at Juntendo University, Japan [14]) are temperature-sensitive *Escherichia coli-S. aureus* shuttle vectors. The plasmid pBT2 contains a chloramphenicol resistance gene, and pSR2 contains a tetracycline resistance gene and *ccr*AB2 genes.

### 2.2. Construction of USA300 arcA and ACME Deletion Mutants

To delete each of *arc*A and ACME in the WT 2406 strain, allelic replacement was performed using pBT2. Briefly, DNA fragments of ~1 kb on the left and right flanking regions of both the *arc*A and ACME loci were amplified by PCR from the genomic DNA of strain 2406. A complete and functional gentamicin resistance cassette was amplified by PCR from M92. The sequences of the primers used are listed in Table 1. The left fragment was digested with SalI and AttII; the right fragment was digested with ClaI and XhoI; and the gentamicin resistance cassette was digested with AttII and ClaI. The pBT2 plasmid which was digested with SalI and XhoI was ligated with all the digested PCR fragments, resulting in the chimeric plasmids, pBT2Δ*arc*A and pBT2ΔACME, each of which contained a chimera with the gentamicin cassette in the middle and the left and right flanking regions in both left and right sides, respectively. Both plasmids were transformed (by electroporation) sequentially into *E. coli* DH5α, *S. aureus* RN4220 and finally the 2406 strain. Allelic replacement of the *arc*A and the ACME genes by the gentamicin gene in the 2406 strain was achieved by growth in brain heart infusion (BHI) with chloramphenicol (20 μg/mL) at 42 °C and then at 30 °C with gentamicin (20 μg/mL). The transconjugants were selected using TSA plates with gentamicin (20 μg/mL). The putative 2406 *arc*A and ACME deletion mutants, Δ*arc*A and ΔACME, were screened for chloramphenicol (20 μg/mL) sensitivity to confirm the loss of the plasmid. The absence of either *arc*A or ACME in the mutants were verified by Pulsed-field gel electrophoresis (PFGE) [15], USA300/USA400 multiplex PCR assay [16] and sequencing using primer arcA-ExtF and USA300-ExtF, which are located outside *arcA* and ACME genes.

### 2.3. Gene Expression in WT and Mutant Strain

Bacterial RNA was isolated using TRIzol and treated with Turbo DNA-free (Invitrogen, Carlsbad, CA, USA) to remove contaminated DNA. RNA was then converted to cDNA using cDNA synthesis kit (BioRad, Hercules, CA, USA). Gene expression of *arcA* and *opp3C* between WT and mutants was compared by RT-PCR using SsoFast EvaGreen Supermix (BioRad), and housekeeping gene *gyrB* was used as an internal control. Primers for RT-PCR are listed in Table 2.

### 2.4. Bacterial Growth in Acidic Environment

Overnight, BHI bacterial cultures were diluted 1:1000 in either BHI broth or BHI supplemented with 1% arginine in different pH values (pH 4–7), and 200 μL of the culture was loaded into a 96-well plate. Each well was covered with 30 μL mineral oil. Plates were incubated at 37 °C and the OD_600_ values were measured every 20 min using a Wallac Victor2 multilabel counter (Perkin Elmer, Waltham, MA, USA).

For growth competition in pH 4.5, one of the competitors was the 2406 WT strain, and the other strain was either Δ*arc*A or ΔACME. The two competing strains were first grown separately for 1 day in BHI and both competitors were in similar physiological states, stationary phase. The two competitors were adjusted to an OD_600_ value of 2.0, and then re-suspended in sterile saline. Equal volumes of bacterial suspension for each competitor (total ~10^4^ CFU/mL, as determined by plate count) were inoculated into fresh BHI (pH 4.5) and were incubated with shaking (200 rpm) at 37 °C for 2 days. The cell densities of the two competing strains were determined by plating on TSA and on TSA with gentamicin (20 μg/mL). The relative fitness (W) of the competitors was calculated as the log ratio of the realized growth rates [17]. Briefly, let N_WT_(0) and N_Δ_(0) be the initial densities of two competitors, WT and mutant respectively, and let N_WT_(1) and N_Δ_(1) be the corresponding densities after 1 day i.e., W_WT,Δ_ = ln[N_WT_(1)/N_WT_(0)]/ln[N_Δ_(1)/N_Δ_(0)]. If the relative fitness (W_WT,Δ_) is greater than 1, WT has better fitness than the mutant; if W_WT,Δ_ is less than 1, the mutant has better fitness than the WT. The experiments were performed in replicates of 11 for Day 1 survival experiment (*n* = 11), and 15 for Day 2 survival experiments (*n* = 15). Mann–Whitney tests were performed to determine significant difference between the W_WT,Δ_ and the value 1.

### 2.5. Bacterial Survival on a Plastic Surface

The competition experiment was used to compare the bacterial survival on a plastic surface (plastic bottom surface of 48-well plate). Two strains (WT and Δ*arcA*, or WT and ΔACME) were mixed in a 1:1 ratio (total ~10^7^ CFU/mL) and were spotted on the bottom of each well of a 48-well plate (Nunc) and then air dried. On Day 1 and 2 post-inoculation, the surviving bacteria were recovered by soaking the wells with sterile saline for 30 min. The bacterial suspensions then were enumerated by serial dilution and plating. After 24 h of incubation, on a culture plate with countable colonies, which was a mixture of WT and mutant colonies, the number of WT or mutant cells in the mixed culture was determined by re-streaking single colonies on TSA with gentamicin, as the WT was sensitive to gentamicin but the mutant was resistant to gentamicin. The ratio of competitors in the mix solution on Day 0 and on the day that bacterial cells were recovered was determined by the number of WT divided by the number of mutant cells (WT/mutant). The competition index (CI), which is defined as the output ratio (WT/mutant) divided by the input ratio [18], was calculated. The experiments were performed in replicates as follows: 15 for WT vs. Δ*arc*A and 21 for WT vs. ΔACME. Student *t* tests were performed to determine significant differences between the CI and the value 1.

### 2.6. Mouse Skin Survival Model

Female BALB/c mice, aged 8–10 weeks, obtained from Charles River, US, were housed at the Faculty of Medicine Animal Care Facility, University of Calgary. The mice were shaved at their back (1 cm^2^) with electrical clippers prior to bacterial inoculation. The skin was wiped with 70% alcohol followed by saline, then dried with Q-tip. For survival competition, equal volumes of the WT and the mutant strains were mixed. This mixed culture was spotted (total ~10^7^ CFU in 4 μL) on the surface of the skin and air dried. The mice were housed individually and were monitored every day. On Days 1, 2 and 4, the skin surface was wiped with Q-tip soaked with sterile saline. The Q-tip was then soaked in 1 mL of sterile saline for 30 min. The bacterial number obtained from the skin was determined by serial dilution and plating on TSA. The recovered colonies were confirmed by PCR, and the WT/mutant strains were enumerated by streaking single colonies on TSA with gentamicin. The competition index of survival was calculated as described earlier. The experiment was done in triplicate, with seven mice included for Days 1, 2 and 4 in each experiment. Mann–Whitney tests were performed to determine significant difference between the CI and the value 1. 

### 2.7. Invasion, Proliferation and Cytotoxicity Assays

**Figure 1:**
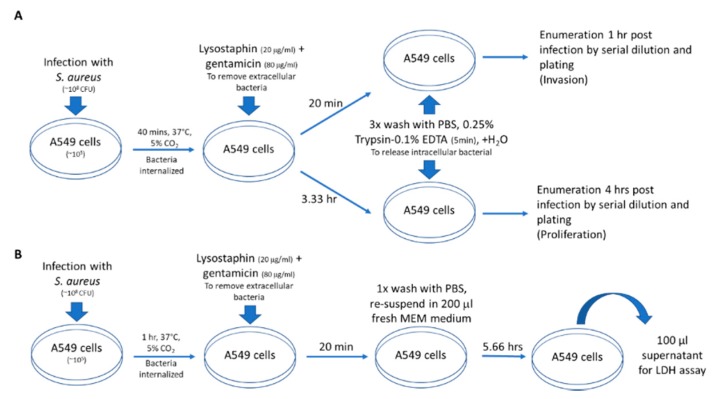
Flow chart for Staphylococcal invasion, proliferation and cytotoxicity assays. (**A**) Invasion/proliferation assay: A549 cells (human lung epithelial cell line) were infected with *S. aureus*, then extracellular bacteria were removed. Cells were washed and lysed then invasion and proliferation assessed at 1 and 4 h post infection, respectively. (**B**) Cytotoxicity assay: A549 cells were infected with *S. aureus*, then extracellular bacteria were removed. The cells were washed and at 7 h post infection supernatant was used in the LDH assay for determining cellular cytotoxicity.

A549 cells, a human lung epithelial cell line, were maintained in minimum essential medium (MEM, Gibco) supplemented with sodium carbonate and 10% fetal bovine serum (FBS, Invitrogen). A549 cells (~10^5^ cells) were seeded into 24-well tissue culture plates (Nunc) (37 °C, 5% CO_2_) 18 h prior to the experiments. One hour prior to the experiments, cells were washed once with PBS, followed by the addition of 1 mL of fresh medium. *S. aureus* (total ~10^8^ CFU) that were harvested at the mid-exponential phase of growth (OD_600_ = 0.7) were inoculated into each well of A549 cells and incubated for 40 min at 37 °C, 5% CO_2_. The extracellular bacteria were removed by treating with lysostaphin (20 µg/µL) and gentamicin (80 µg/µL) for 20 min. The monolayer was washed three times with PBS, treated with 200 μL of 0.25% trypsin-0.1% EDTA for 5 min at 37 °C, and lysed by the addition of 800 μL of cold sterile distilled water to release intracellular staphylococci. The intracellular bacteria at 1 h (bacteria internalization) and 4 h (bacterial proliferation) were enumerated by serial dilution and plating. For the cytotoxicity assay, the A549 cells inoculated with bacterial cells were incubated at 37 °C for one hour, and then were treated with gentamicin and lysostaphin. After the treatment, the cells were washed once with PBS and then re-suspended in fresh MEM medium (200 µL). At 7 h, 100 µL of the cell supernatant from each well were aliquoted for lactate dehydrogenase (LDH) assay (Roche Applied Science, Mannheim, Germany) in triplicate. CMRSA7 was used as a low cytotoxicity control strain because it consistently exhibited low cytotoxicity in A549 cells. A summary flow chart detailing the procedure is presented in Figure 1. Student *t* tests were performed to determine significant differences between the tested strains.

### 2.8. Caenorhabditis elegans and Drosophila melanogaster Killing Assays

The *C. elegans* killing assay was described previously [19]. Briefly, bacteria were grown on TSA plates supplemented with 5 μg/mL nalidixic acid (NA), followed by addition of 30 L4-stage nematodes per plate, incubation at 25 °C, and scoring for live and dead worms every day for 7 days.

*D. melanogaster* Canton S were maintained at room temperature on standard cornmeal agar. The *D. melanogaster* killing assay (pricking assay) was performed as described previously [20]. Briefly, female flies (2–5 days old) were injected in the dorsal thorax with a 27-gauge needle dipped directly into *S. aureus* bacterial suspension (8 × 10^8^ CFU/mL). The live and dead flies were monitored at room temperature for 4 days.

Survival curves for both *C. elegans* and *D. melanogaster* models were generated by the Kaplan–Meier method, and statistical significance was calculated by log-rank test using Prism 5 (GraphPad Software, Inc., San Diego, CA, USA).

### 2.9. Mouse Skin Intradermal Infection Model

Female BALB/c mice (*n* = 17), aged 6–8 weeks were housed in groups of 5. Mice had their backs shaved with electrical clippers in advance of inoculation. Fifty (50) µl of bacterial cell suspension (10^8^ CFU/mL) in sterile saline were injected intradermally. The mice were housed individually after inoculation and abscess formation was monitored daily for 14 days. On Day 4, 7 and 14 post-inoculation, the mice were euthanized and skin samples were harvested for histological examination (Animal use protocol approval in accordance with the Guidelines of the Canadian Council on Animal Care by the Health Science Animal Care Committee, University of Calgary). Five mice were included for each group for the initial experiment, and 12 were include for each group for the repeat experiment. Lesion sizes were measured during the repeat experiment and the area (length × width) calculated. Student *t* tests were performed to determine if there was significant difference between WT and mutant lesion sizes.

### 2.10. Epidemiology of ACME Positive and Negative USA300 Clinical Isolates

All clinical isolates from patients infected and/or colonized at the Alberta Health Services (AHS)-Calgary Zone (formerly Calgary Health Region, with ~1.3 million population) who were defined as patients with no previous history of MRSA infection/colonization within a 1-year period prior to bacterial isolation were characterized by PFGE. According to the PFGE pattern, USA300 clinical isolates were identified from the years 2000–2008 (no data from December 2008). Isolates were tested by a USA300/USA400 multiplex PCR assay [16]. Representative isolates were further characterized by SCC*mec* typing [21,22], staphylococcal protein A (*spa*) typing [23], multilocus sequence typing (MLST) [24], and accessory gene regulator (*agr*) typing [25]. The identification of MRSA isolates as USA300 was based on the similarity of PFGE patterns to the USA300 control strains, SCC*mec* type IVa, *spa* type t008, MLST type ST8, *agr* type I and the presence of PVL with or without the ACME cassette. ACME positive and negative (ACME^+^/^−^) USA300 clinical isolates were differentiated by the shifting of the band containing ACME on the PFGE pattern, and were further confirmed by a USA300/USA400 multiplex PCR assay [16]. The incidence of ACME^+^/^−^ isolates was compared yearly from 2000 to 2008.

ACME^+^/^−^ clinical isolates were separated into two categories based on the anatomic sites from where they were taken, which included clinically invasive sites (blood, wound, sputum, bone, graft, sterile fluid, urine and eye) and colonization sites (nose, rectum, vagina, skin). For analysis of contingency data derived from the two groups, Fisher’s exact test was used.

### 2.11. Excision of ACME and/or SCCmec Catalyzed by Recombinase CcrAB2

Strain 2406 was transformed with the plasmid pSR2 containing a *ccr*AB2 gene complex. Strain 2406-pSR2 was grown in BHI broth at 30 °C followed by incubation at 42 °C, and then plated on TSA plates to obtain single colonies, which were screened for excision of ACME, SCC*mec* and ACME-SCC*mec* by PCR. All the PCR primers (X1-X6) used were described previously [10] except that X3’ (5′-GTAACTACGCACTATCATTCAGC-3′) was designed in this study and was used instead of X3. These strains with excision were named as exACME^−^, exSCC*mec*^−^ and exACME^−^SCC*mec*^−^.

### 2.12. Spontaneous Excision of ACME and/or SCCmec

The frequency of spontaneous excision/circularization of ACME and/or SCC*mec* in the 2406 strain or other *S. aureus* strains were determined by qRT-PCR using the primer pairs of X1-X6. The frequency of excision/circularization was calculated as the gene copy of excision/circularization (Ct of each primer pair XnXn) relative to the total gene copy number, which was represented by Ct of a housekeeping gene *gyr*B. The frequency of excision is roughly calculated as 10^−(Ct XnXn − Ct *gyr*B)/3.32^, because the Ct value is ~3.32 apart every 10-fold dilution in the standard curve.

To screen for spontaneous ACME and/or SCC*mec* deletion, overnight, a USA300-2406 bacterial culture was serially diluted and plated on TSA plates, and 800 single colonies were screened for exSCC*mec*^−^ and exACME^−^SCC*mec*^−^ strains on TSA plates with 4 µg/mL oxacillin because they were sensitive to oxacillin without SCC*mec*. Alternatively, an overnight USA300-2406 bacterial culture was adjusted to OD_600_ 2.0 and then re-suspended in sterile H_2_O. A bacterial suspension (100 µL) was spotted in a single well of a 24-well plate. After 2 days at room temperature, live bacteria were recovered by soaking the well with 1 mL sterile saline, and then, serial dilution and plating on TSA plates. After 24 h, 450 single colonies were screened on TSA plates with 4 µg/mL oxacillin. Colonies containing ACME deletion were screened by the USA300/USA400 multiplex PCR assay [16], which can detect the presence/absence of *mec*A and *arc*A genes.

### 2.13. Ethics Approval

The protocol for animal use was approved by the Animal Care Committee, University of Calgary (Protocol #: M06074, M09115, AC13-0076 and AC17-0241, approved on Dec. 4, 2008, Dec. 18, 2009, Jan. 13, 2017, and May 3, 2018, respectively).

## 3. Results

### 3.1. Construction of USA300 arcA and ACME Deletion

Isogenic ACME and *arc*A (a key gene in the arginine deiminase pathway) mutants of USA300-2406, were created by allelic replacement. The ACME genomic island or the *arcA* gene was replaced with a gentamicin resistance gene to generate ΔACME and Δ*arc*A mutants.

**Figure 2:**
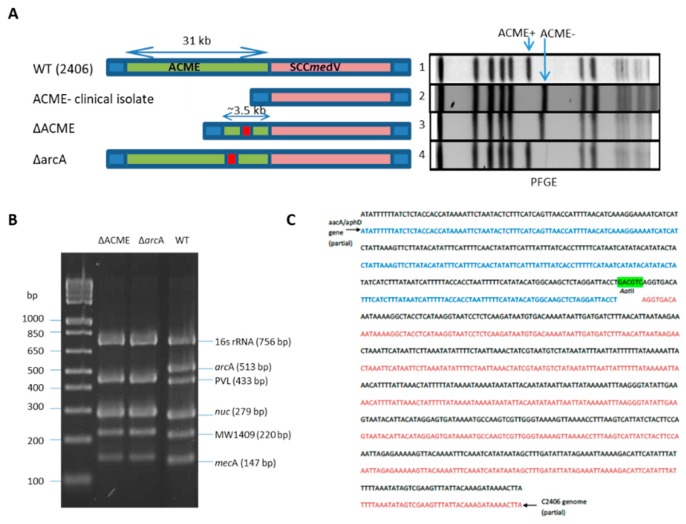
Construction of USA300 *arc*A and ACME deletion mutants. (**A**) Confirmation of ΔACME and Δ*arc*A by PFGE. Left panel is a diagram of ACME-SCC*mec* regions on the bacterial chromosomes of ACME^+/−^ USA300 clinical isolates, ΔACME and Δ*arc*A. The right panel is the PFGE patterns of the following strains: Lane 1, ACME^+^ USA300 clinical isolate (2406 WT); 2, ACME^−^ USA300 clinical isolate; 3, ΔACME; 4, Δ*arc*A. Arrows indicate the fragment containing ACME in WT, while a band shift is observed for the ACME^−^ strains. (**B**) ΔACME and Δ*arc*A confirmation by a USA300/USA400 muliplex PCR assay: *arc*A, a USA300 specific gene, is present in WT, but missing from ΔACME and Δ*arc*A. Lane M, 1 kb Plus DNA ladder (Invitrogen). (**C**) The junction area of genome and antibiotic resistant gene was sequenced and aligned with *aac*A/*aph*D gene and C2406 genome in NCBI database. The black sequence is junction sequence expected to span *aac*A/*aph*D gene and C2406 genome, the blue is partial *aac*A/*aph*D gene and the red is partial C2406 genome sequence. The sequence highlighted with green is the AatII restriction site used for cloning.

As shown in Figure 2A, PFGE confirmed that ACME (31 kb) was successfully deleted from the chromosome in ΔACME according to the shift of the band containing ACME compared with WT (lane 1 and 3). USA300/400 Multiplex PCR assay, detecting the presence/absence of 6 genes, of which *arc*A gene is specific for USA300 strain, further confirmed the deletion of *arc*A gene in both ΔACME and Δ*arc*A mutants (Figure 2B) [16]. In addition, the mutants were confirmed by direct sequencing of the chromosomal DNA and the sequence alignment of junction area of genome and inserted antibiotic resistant gene was shown in Figure 2C. Gene sequencing confirmed that left and right flanking regions of target genes in the WT were ligated to the gentamicin cassette in the deletion mutants.

To further confirm that ACME and *arc*A were absent in the mutants, the expression of *arc*A and *opp-3*C were compared between WT, ΔACME and Δ*arc*A. The results showed that *arc*A was expressed in WT in vitro condition, but not in ΔACME and Δ*arc*A. For *opp-3*C, it was also expressed in WT and in a much less extent in Δ*arc*A, but not in ΔACME.

### 3.2. Growth Difference among Strains 2406 WT, ΔarcA and ΔACME in Acidic Environment

**Figure 3:**
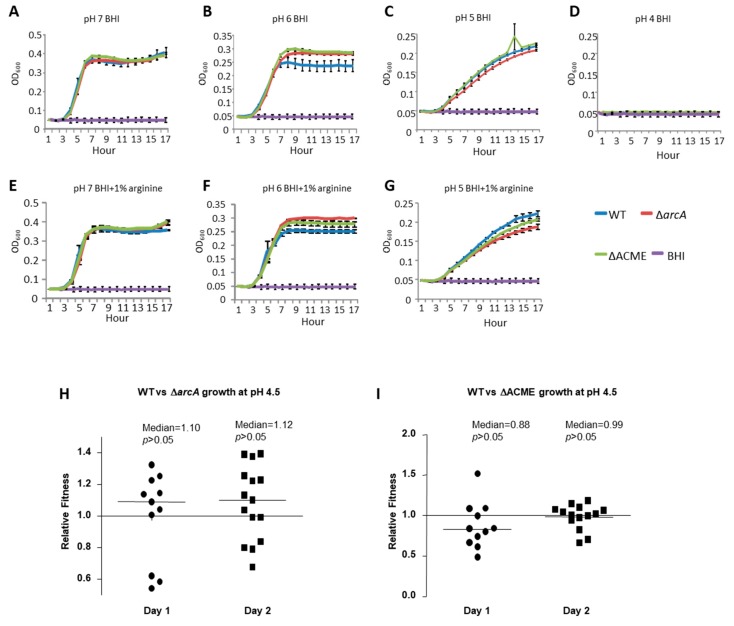
Growth of strain 2406 WT, Δ*arc*A and ΔACME in acidic environment. OD_600_ was plotted over time for growth of the strains in media of differing pH. (**A**–**G**): WT, Δ*arc*A, ΔACME growth curves in BHI at pH 7 (**A**), pH 6 (**B**), pH 5 (**C**), pH 4 (**D**), pH 7+1% arginine (**E**), pH 6+1% arginine (**F**), and pH 5+1% arginine (**G**). No significant difference was observed between WT and mutants (Δ*arc*A and ΔACME). The blue curve represents the WT strain, red represents Δ*arc*A, green represents ΔACME, and purple represents the BHI control without bacteria. (**H**,**I**): WT vs. Δ*arc*A (**H**) and WT vs ΔACME (**I**) competition experiments in BHI (pH 4.5). No difference observed between WT and mutants, as the means of W_WT,__Δ_ were not significantly different from 1 on Day 1 and Day 2, respectively. Mann–Whitney tests were performed to determine significant difference between the W_WT,Δ_ and the value 1. The bar indicated the median value of the relative fitness.

The arginine deiminase pathway has been demonstrated to enhance bacterial tolerance in a low pH environment by producing ammonia **[5]**. To investigate whether ACME, specifically the arc gene cluster, may promote bacterial growth in an acidic environment, the growths of the WT, Δ*arc*A and ΔACME strains in pH 7, 6 and 5 with or without arginine (1%) in culture broth were compared. As shown in Figure 3A–G, the growth rates and the maximum densities for 2406 WT, Δ*arc*A and ΔACME decreased as pH decreased. For bacterial growth in the culture broth with or without arginine, at pH 7, the growth curves for WT, Δ*arc*A and ΔACME were almost identical (Figure 3A,E); at pH 6, these strains showed similar growth rates, but the maximum density of the WT was lower than that of the mutants (Figure 3B,F); at pH 5, no typical log phase was observed for all three strains, and a slight difference was observed among the growth curves, with Δ*arc*A showing slightly lower growth rate and maximum density than the WT and ΔACME (Figure 3C,G); at pH 4, no growth was observed for all strains (Figure 3D). No significant difference was observed between the WT and mutants (Δ*arc*A and ΔACME).

To determine whether the small differences among WT, Δ*arc*A and ΔACME shown in the growth curves indicates slower growth in an acidic environment, a more sensitive competition experiment was used to compare WT vs. Δ*arc*A and WT vs. ΔACME growth in BHI (pH 4.5). A pH 4.5 was chosen to maximize the difference between the WT and the mutants, because no bacterial growth was observed at pH 4, but the difference between the WT and the mutants was too small at pH 5. On Day 1 and Day 2, the relative fitness for WT vs. Δ*arc*A and WT vs. ΔACME ranged from 0.5 to 1.4, and the median (0.88–1.12) were close to 1 (Figure 3H,I), no significant difference was observed between the relative fitness and 1 (*p* > 0.05).

### 3.3. The Survival Ability of Strain 2406 WT, ΔarcA and ΔACME on the Different Surfaces

*S. aureus* can be spread by human contact of *S. aureus* contaminated surfaces. Since *S. aureus* can survive on dry contaminated surfaces for months, inanimate objects can therefore continue to be a source for *S. aureus* transmission [26]. Thus, it is possible that greater persistence in the environment may contribute to the wider spread of USA300. To test the possible role of ACME or *arc* in enhancing the persistence of USA300 in the environment, the survival curves of 2406 WT, Δ*arc*A and ΔACME on the surface of wood, paper, and plastic for 3 days were compared, and no significant difference between these strains was observed in terms of the survival rate on those surfaces (data not shown).

**Figure 4:**
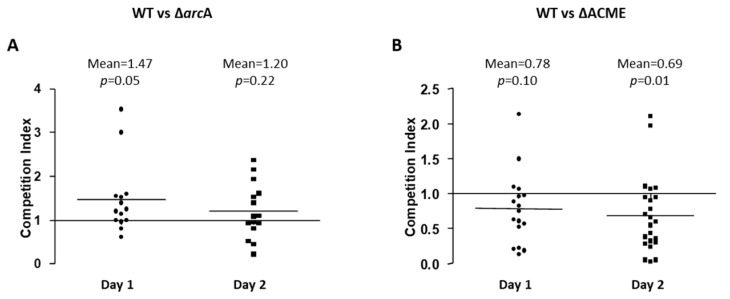
Survival of strain 2406 WT, Δ*arc*A and ΔACME on the surface of plastic. WT vs. Δ*arc*A (**A**) and WT vs. ΔACME (**B**) strains were mixed in equal proportions and survival competition experiments on plastic surface done. CIs of WT vs. Δ*arc*A and WT vs. ΔACME indicated that ACME may not play a role in the bacterial survival on the plastic surface, with ΔACME showing lower death rates than WT. Student *t* tests were performed to determine significant differences between the CI and the value 1.

To avoid sample to sample variation, a more sensitive competition experiment was used to compare the survival ability of WT, Δ*arc*A or ΔACME on the plastic surface. On Day 1 and 2, the survival competition indexes of WT vs. Δ*arc*A or WT vs. ΔACME were determined (Figure 4A,B). The means of CIs of WT vs. Δ*arc*A on Day 1 (mean = 1.47) and 2 (mean = 1.2) were greater than 1, but no significant difference was observed (*p* ≥ 0.05); the means of CIs of WT vs. ΔACME on Day 1 (mean = 0.78) and 2 (mean = 0.69) were less than 1, and significant difference was observed on Day 2 (*p* = 0.01).

### 3.4. The Survival Ability of Strain 2406 WT and ΔACME on the Surface of Mouse Skin

**Figure 5:**
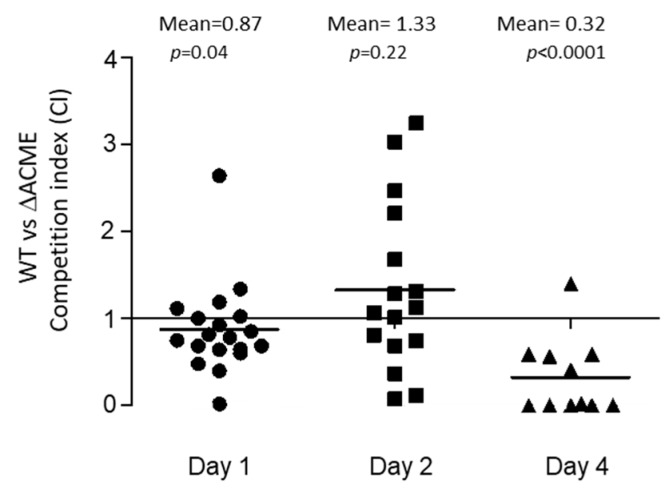
Strain 2406 WT vs. ΔACME competition experiments showing survival on the surface of mouse skin. The mean CIs for WT vs. ΔACME on mouse skin was 0.87 on Day 1 (*p* = 0.04), 1.33 on Day 2 (*p* = 0.22) and 0.32 on Day 4 (*p* < 0.0001), indicating that ΔACME had slow death rates on the surface of mouse skin. Mann–Whitney tests were performed to determine significant difference between the CI and the value 1.

*S. aureus* may be spread by skin–skin contact, and ACME was hypothesized to contribute to bacterial survival on the acidic human skin environment (pH 4.2–5.0) [4]. Here, a competition experiment was used to compare the survival ability of WT and ΔACME on the surface of mouse skin, an indicator for bacterial transmissibility. Starting with an average concentration of 5.2 × 10^10^ CFU/mL of WT and ΔACME (1:1 mixed), bacterial suspension were spotted on the mouse skin. An average of 3.7 × 10^4^ CFU/mL of bacteria was recovered on Day 1, while an average of 4.4 × 10^3^ CFU/mL was recovered on Day 2, post-inoculation. On Day 4 post inoculation, recovery ranged from 0–77 CFU/mL. The mean CI on Day 1 was 0.87 (CI vs. the value 1, *p* = 0.04), while on Day 2 it was 1.33 (*p* = 0.22), and on Day 4 the mean was 0.32 (*p* < 0.0001) (Figure 5). The mean CI on Days 1 and 4 were below 1, suggesting that the WT is less competitive than ΔACME on the mouse skin surface (Figure 5). Of note, on Day 4 too few or no bacteria were recovered from 10 out of 21 mice, however, in the other 11 mice where bacteria were recovered ΔACME predominated (CI mean = 0.32). Therefore, the competition experiments suggested that ΔACME had a slower death rate (Day 1 and 4) than the WT strain in terms of survival on the surface of mouse skin.

### 3.5. Strain 2406 WT and ΔACME Showed Similar Invasion, Intracellular Replication and Cytotoxicity in the Human Lung Epithelial Cell (A549)

**Figure 6:**
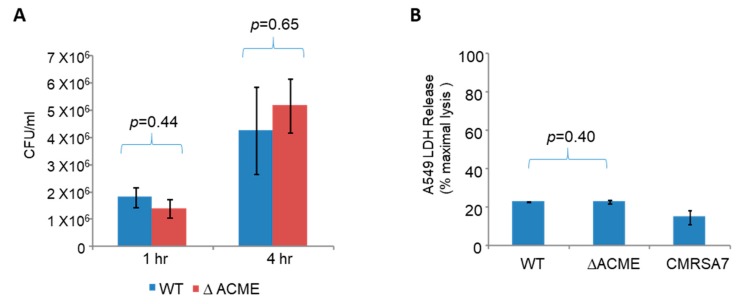
Strain 2406 WT and ΔACME showed similar invasion, intracellular replication and cytotoxicity in the human lung epithelial cell (A549). (**A**) WT and ΔACME showed similar ability of invasion and replication insideA549 cells. The number of intracellular bacteria at 1 h was indicative of bacterial invasion ability, while at 4 h was indicative of bacterial replication ability. (**B**) WT and ΔACME showed similar cytotoxicity in A549 cells at 7 h post-infection. The cytotoxicity was determined by the LDH release induced by bacteria relative to maximal cell lysis. CMRSA7 was included as a low cytotoxicity control because it consistently exhibited low cytotoxicity. Student *t* tests were performed to determine significant difference between the tested strains.

*S. aureus* is capable of invading and replicating within epithelial cells [27,28], and it was hypothesized that ACME may enhance the *S. aureus* survival inside an acidic intracellular environment. As ΔACME and Δ*arc*A did not show significant difference in the survival assays described above, only ΔACME was chosen for further virulence studies. To investigate the role of ACME in the intracellular survival of *S. aureus*, bacterial invasion, intracellular replication and cytotoxicity were determined using human lung epithelial cells. As shown in Figure 6A, the average number of invading bacteria was 1.8 × 10^6^ and 1.4 × 10^6^ CFU/mL for the WT and ΔACME, respectively, at 1 h after the monolayer was incubated with ~10^8^ CFU bacteria. At 4 h post-infection, the average number of intracellular bacteria increased to as much as 4-fold (4.5 × 10^6^ and 5.2 × 10^6^ CFU/mL for the WT and ΔACME, respectively), indicating that *S. aureus* can replicate inside the cell. However, there was no significant difference between the WT and ΔACME (*p* > 0.45). The cytotoxicity of the WT and ΔACME was determined 7 h post-infection. As shown in Figure 6B, the WT and ΔACME both induced 22.7% and 22.8% LDH release relative to maximal cell lysis, while CMRSA7, a low cytotoxicity control, only induced 13.9% LDH release. There was no difference observed between WT and ΔACME for the cytotoxicity (*p* = 0.4).

### 3.6. Strain 2406 WT and ΔACME Showed Similar Virulence in Invertebrate Models

**Figure 7:**
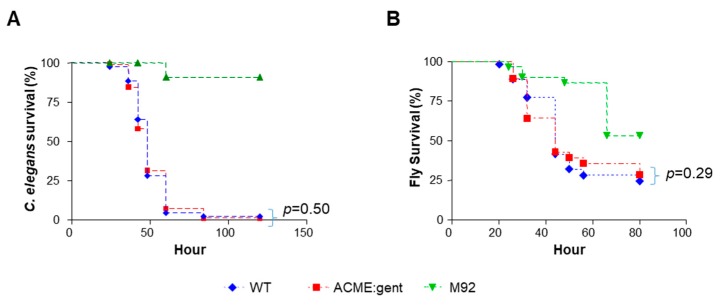
Strain 2406 WT and ΔACME showed similar virulence in the *C. elegans* and the *D. melanogaster* model. (**A**) Kaplan–Meier survival plots of nematodes fed with WT, ΔACME, and M92 (negative control). The survival curves of WT and ΔACME are not significantly different (*p* = 0.50); (**B**) Kaplan–Meier survival plots of flies infected with WT, ΔACME and M92 (negative control). The survival curves of WT and ΔACME are not significantly different (*p* = 0.92). Statistical significance was calculated by log-rank test.

To investigate whether ACME contributes to the virulence of USA300-2406, the WT and ΔACME strains were analyzed with the *C. elegans* and *D. melanogaster* killing assays. Both WT and ΔACME demonstrated killing activity in *C. elegans* and *D. melanogaster* models. In the *C. elegans* model, 68.6% and 71.9% worms at 48 h, and 98.8% and 97.8% worms after 72 h were killed by the WT and ΔACME, respectively. No difference was observed between WT and ΔACME (*p* = 0.50) (Figure 7A). In the *D. melanogaster* model, 67.9% and 60.7% flies at 48 h, and 75.5% and 71.4% flies after 72 h were dead for WT and ΔACME, respectively (Figure 7B). No difference was observed between WT and ΔACME in the *D. melanogaster* model (*p* = 0.29).

### 3.7. Strain 2406 WT, ΔACME and ΔarcA Showed Similar Virulence in a Mouse Skin Infection Model

**Figure 8:**
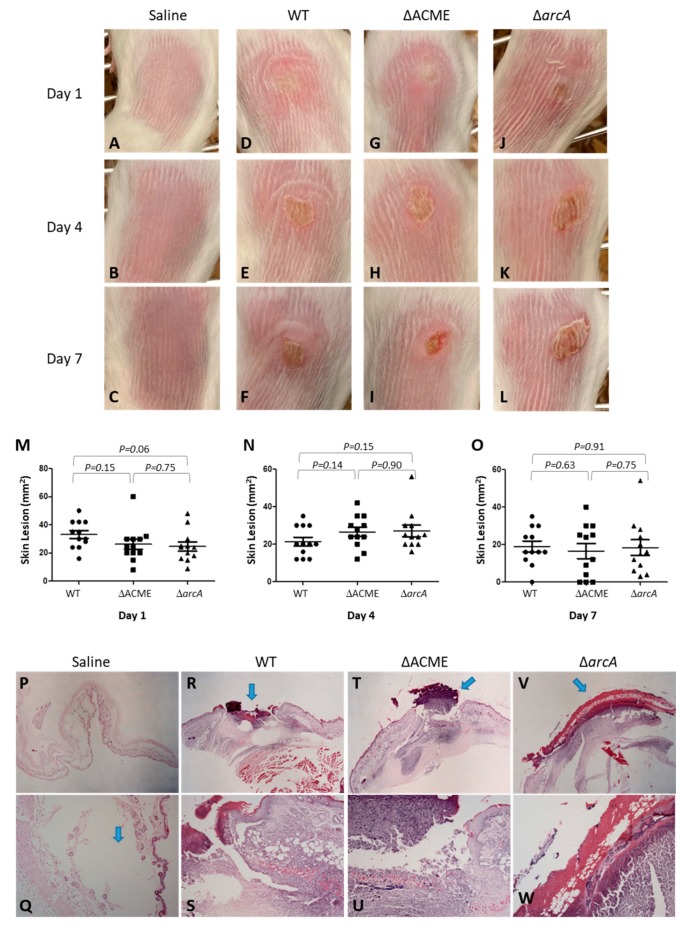
Representative images of lesions formed following USA300 intradermal infection of BALB/c mice. (**A**–**C**) Mice injected with saline were used as negative control on Day 1, Day 4 and Day 7. (**D**) Mice infected with the wild type strain on Day 1, (**E**) Day 4 and (**F**) Day 14; (**G**) Mice infected with ΔACME on Day 1, (**H**) Day 4, and (**I**) Day 7; (**J**) Mice infected with Δ*arc*A on Day 1, (**K**) Day 4, and (**L**) Day 7. No differences were noted between the groups on each day. (**M**–**O**) Lesion sizes (as measured by total area mm^2^) for mice infected with WT (2406), ΔACME and Δ*arc*A are shown, for Days 1, 4 and 7. No statistical significance was noted between WT and either of the mutants, on any of the days (all *p* > 0.05). Day 1: the mean lesion sizes for mice infected with WT, ΔACME and Δ*arc*A were 33.17, 26.25 and 24.67 mm^2^, respectively; Day 4: 21.42, 26.58 and 27.08 mm^2^ for WT, ΔACME and Δ*arc*A, respectively; Day 7: 19.00, 16.58 and 18.42 mm^2^ for WT, ΔACME and Δ*arc*A, respectively. (**P**–**W**) Histopathology examination of skin lesions caused by WT and ΔACME: Top panel is low magnification (×10), and bottom panel is high magnification (×40). P and Q, negative control (saline), the arrow indicates the injection site; R and S, WT induced focal ulceration (arrow) and supurative dermatitis/panniculitis; T and U, ΔACME induced the same focal ulceration (arrow) and supurative dermatitis/panniculitis; V and W, Δ*arc*A induced locally extensive and necro-suppurative dermatitis/panniculitis (arrow).

To determine whether the ACME and specifically the arginine deiminase pathway of strain USA300 contributes to the bacterial skin infection, WT, ΔACME and Δ*arc*A were tested in a mouse skin intradermal infection model. Skin abscess formation in mice was monitored after infection. Skin abscesses formed on Day 1 after inoculation and it developed into an open wound from Day 4 to Day 7. The wound started to heal after 7 days and almost recovered on Day 14. The representative pictures of the skin abscess among mice tested for each strain on Day 1, 4 and 7 are shown in Figure 8A–L. During the 14-day experiment period, no difference was observed between the WT, ΔACME and Δ*arc*A in terms of abscess appearances and open wound formation. No significant differences were noted in lesion sizes between mice infected with WT vs. ΔACME and Δ*arc*A, or ΔACME vs. Δ*arc*A (Figure 8M–O). On Day 1, the mean lesion sizes for mice infected with WT, ΔACME and Δ*arc*A were 33.17, 26.25 and 24.67 mm^2^, respectively, with *p* = 0.15 (WT vs. ΔACME), *p* = 0.06 (WT vs. Δ*arc*A), and *p* = 0.75 (ΔACME vs. Δ*arc*A). On Day 4, the mean lesion sizes for mice infected with WT, ΔACME and Δ*arc*A were 21.42, 26.58 and 27.08 mm^2^, respectively, with *p* = 0.14 (WT vs. ΔACME), *p* = 0.15 (WT vs. Δ*arc*A) and *p* = 0.90 (ΔACME vs. Δ*arc*A). On Day 7, the mean lesion sizes for mice infected with WT, ΔACME and Δ*arc*A were 19.00, 16.58 and 18.42 mm^2^, respectively, with *p* = 0.63 (WT vs. ΔACME), *p* = 0.91 (WT vs. Δ*arc*A) and *p* = 0.75 (ΔACME and Δ*arc*A). This result correlated with the histopathology examination. Both WT and ΔACME induced focal ulceration and dermatitis/panniculitis (moderate or severe) in the mouse model, and Δ*arc*A induced locally extensive and necro-suppurative dermatitis/panniculitis (moderate to severe) (Figure 8P–W).

### 3.8. Epidemiology Features of ACME^+^/^−^ USA300 Clinical Isolates

**Figure 9:**
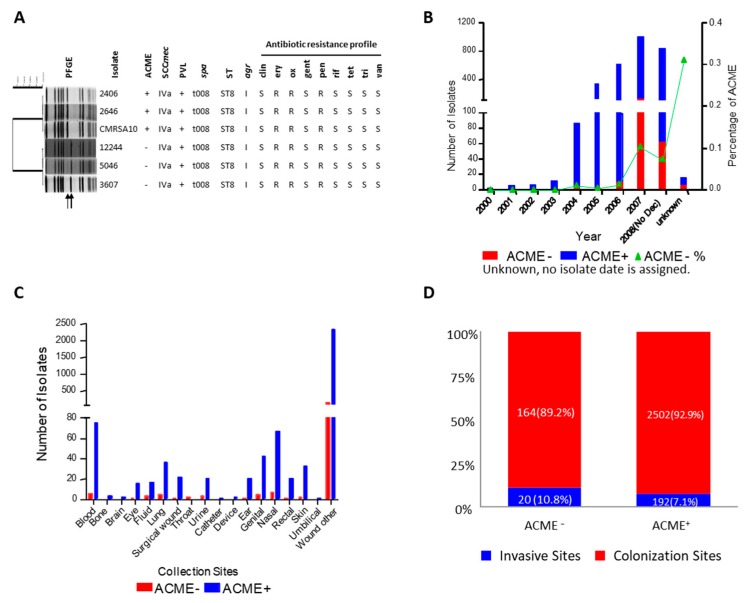
Epidemiological features of USA300 ACME^+^/^−^ clinical isolates in AHS-Calgary. (**A**) PFGE fingerprint, genotypic and phenotypic characteristics of ACME^+^/^−^ USA300 clinical isolates showing that ACME^+^ and ACME^−^ clinical isolates shared the same genotypic and phenotypic features, with the exception of ACME (PFGE band containing ACME is indicated by arrows). 2406 and 2646 are two representative ACME^+^ USA300 clinical isolates; CMRSA10 is a USA300 reference strain provided by the National Microbiology Laboratory; 12244, 5062, and 3607 are three representative ACME^−^ USA300 clinical isolates. PVL (+, positive; −, negative); antibiotic resistant profile (S, sensitive; R, resistant); clin, clindamycin; ery, erythromycin; ox, oxacillin; gent, gentamicin; pen penicillin; rif, rifampicin; tet, tetracycline; tri, trimethoprim-sulfamethoxazole; van, vancomycin. (**B**) The rapid increase of newly diagnosed cases of USA300 infection/colonization cases in AHS-Calgary during 2000–2008. Unknown: no collection date was assigned. ACME^+^ are represented by blue bars, ACME^−^ by red bars, and the green line indicates the increase of ACME^−^ isolates since 2004. (**C**) ACME^+^/^−^ clinical isolates collected from different anatomic sites, with most coming from skin wound, but also some invasive sites. ACME^+^ are represented by blue bars, ACME^−^ by red bars (**D**) ACME^+^/^−^ isolates were not different in isolation from invasive anatomic sites (10.8% for ACME^−^ vs. 7.1% for ACME^+^ [*p* = 0.1183, by Fisher’s exact test]). Invasive sites are represented by blue bars, and colonization sites by red bars.

During the 8-year period from July 2000 to November 2008, all newly isolated MRSA clinical isolates from AHS-Calgary Zone were typed by PFGE and the other molecular methods, and a total of 2878 isolates were found to be USA300 with or without ACME cassette. Two patterns were identified among these isolates, and they belonged to ACME^+^/^−^ isolates according to the shift of the corresponding ACME-containing fragment on the PFGE pattern and presence/absence of *arcA* (Figure 9A). Although the ACME in ACME^+^/^−^ clinical isolates exhibited different PFGE patterns, all of these isolates carried the same SCC*mec* IVa, belonged to MSLT type ST8 (3-3-1-1-4-4-3), SPA type t008 (YHGFMBQBLO) and *agr* type I (Figure 9A). All isolates were resistant to β-lactams but susceptible to many other antibiotics including tetracycline, rifampicin and vancomycin (Figure 9A).

Despite sharing similar PFGE patterns, genotypic and phenotypic characteristics, 2694 out of 2878 (93.6%) isolates were ACME^+^, and the remaining 184 isolates (6.4%) were ACME^−^. ACME^+^ isolates, which emerged in 2000, increased over time and became dominant after 2004; while ACME^−^ isolates emerged in 2004 and increased over time to date. The percentage of ACME^−^ isolates out of the total USA300 isolates increased every year, from 1.2% in 2004 to 10.5% in 2007, with the percentage being 7.4% from January to November 2008 (Figure 9B).

According to the collection sites, most USA300 clinical isolates (85.5%) were associated with skin wound, while some isolates could be isolated from invasive anatomic sites, such as blood, bone and lung (Figure 9C). When these anatomic sites were grouped into two categories, invasive and colonization sites, ACME^+^ and ACME^−^ isolates showed similar invasive-site frequencies (10.8% for ACME^−^ vs. 7.1% for ACME^+^, *p* = 0.12) (Figure 9D), suggesting that ACME was not associated with more invasive infection.

### 3.9. Spontaneous ACME and/or SCCmec Excision from the Chromosome

As demonstrated in the previous section, ACME^−^ USA300 clinical isolates emerged in 2004 and increased over time in AHS-Calgary Zone. We therefore hypothesized that ACME could be excised from the genome naturally (spontaneously) to generate ACME^−^ strains in a low frequency because there is a native recombinase (*ccr*AB2) gene located inside the *SCCmec* complex in the USA300 genome. To test this hypothesis, we created the following reference strains: exACME^−^, exSCC*mec* and exACME-SCC*mec*, which ACME, SCC*mec* and ACME-SCC*mec* have been excised from the chromosome, respectively, by transforming the plasmid pSR2 carrying recombinase *ccr*AB2 into USA300-2406. We then detected the spontaneous excision of ACME and/or SCC*mec* in strain USA300-2406 by utilizing the high sensitivity and the quantitative feature of qRT-PCR assays. For strain USA300-2406, the frequency of spontaneous excision of ACME from the genome is 10^−5.5^, while the excision frequencies of SCC*mec* and ACME-SCC*mec* from the genome were 10^−3.6^ and 10^−3.7^, respectively (Table 3). Moreover, with different primer combinations, our results showed that ACME, SCC*mec*, or both together (ACME-SCC*mec*) were able to form circularized DNA after spontaneous excision from the chromosome (Table 3).

Spontaneous excision of ACME and/or SCC*mec* in strains exACME^−^ and exSCC*mec*^−^ were also tested. As shown in Table 2, SCC*mec* still could be excised from the chromosome in exACME, with frequency of 10^−2.7^. On the other hand, the spontaneous excision of ACME has not been observed in exSCC*mec*^−^. Moreover, the spontaneous excision of SCC*mec* was also tested in other MRSA strains (as control). In the MRSA strain COL, SCC*mec* I did not spontaneously excise from the chromosome because it has a premature stop codon in *ccr*B gene [29]. In strain CMRSA2, SCC*mec* II was able to excise but the frequency is 10^−6.1^, which is lower than the excision frequency of SCC*mec* IV in USA300-2406 (Table 3).

The circularized DNA inside the bacterial cell could be lost because there is no replication origin in the circularized DNA. To investigate whether these circularized DNA could be lost to generate the natural ACME and/or SCC*mec* deletion strains, about 1250 single colonies were screened from USA300-2406. No excision was identified among 800 colonies from overnight BHI culture broth; while two exACME^−^SCC*mec*^−^ and one exSCC*mec*^−^ colonies were isolated among 450 colonies recovered from the surface of plastic but no exACME^−^ was identified.

## 4. Discussion

The genome sequence of the CA-MRSA predominant strain USA300 has revealed the presence of a unique genomic island, ACME, in USA300, and ACME has been hypothesized to contribute to the enhanced virulence and transmission of USA300 [4,30]. In a rabbit bacteremia model, Diep et al. demonstrated that WT and an SCC*mec* deletion mutant strain (SCC*mec*^−^) have the same fitness but the WT strain shows better fitness than the ACME-SCC*mec* deletion mutant strain (ACME-SCC*mec*^−^), indirectly suggesting that ACME contributes to bacterial fitness [10]. However, USA300 has been mostly associated with skin and soft tissue infection, and sometimes associated with severe infections, such as necrotizing pneumonia [31]. In a rat pneumonia model and a mouse skin infection model, Montgomery et al. compared the same WT and isogenic ACME^−^ strain, as well as ACME^−^ clinical strains and found no difference between WT, isogenic ACME^−^ strain and ACME^−^ clinical strains in terms of survival rates and skin lesion appearances, suggesting that ACME may not be necessary for bacterial virulence in these models [11]. On the other hand, two latter studies suggest that ACME contributes to USA300 persistence in the acidic skin environment and within healing abscesses, which may provide complemental information for the function of ACME in bacterial virulence [9,32]. Both studies show that SpeG, which is encoded by ACME, confers resistance to polyamine, and provides a fitness advantage to USA300 during SSTI. However, Thurlow’s study also shows that WT USA300, isogenic ΔACME and Δ*arc*Δ*speG* mutants have similar viable CFU per abscess in a skin infection model, suggesting that *speG* is dispensable without ACME-Arc [9].

In the present study, first, WT, Δ*arc*A and ΔACME, had similar growth rates in acidic environment, different than Thurlow et al.’s study, which has shown that in a chemically-defined medium buffered at pH 5.0 that contains arginine and lactic acid, USA300 WT demonstrates 2 log growth but not ΔACME and ΔArc. The different results may be attributed to different experimental conditions. It is arguable that the defined medium supplement with lactic acid would be close to the skin’s condition. However, the nutritional environment on human skin surface, which is determined by skin cells, skin microbiota, external environment, as well as personal hygiene habits, is much more complicated than the defined medium [33]. Therefore, the culture condition using undefined acidified BHI in the present study may be relatively closer to human skin surface. Furthermore, this study shows that WT, Δ*arc*A and ΔACME have similar survival ability on the surface of different objects, including wood, glass and paper (data not shown), and on the surface of human skin (data not shown), suggesting ACME may not contribute to bacterial survival and transmission.

More interestingly, the competition experiments show that ΔACME even has a slower death rate than WT on the surface of plastic and the surface of mouse skin. It is speculated that the slow death rate of ΔACME may result from the absence of a 31 kb ACME DNA fragment in the chromosome compared with the WT. These results suggest that ACME may not play a major role in USA300 transmission and virulence, which is supported by the epidemiologic data for ACME^+^/^−^ USA300 isolates in our local health setting. ACME^−^ USA300 clinical isolates emerged in 2004 and continued to increase over the years, suggesting that ACME is not important for transmission. Also, the ACME^−^ USA300 clinical isolates were similar to ACME^+^ clinical isolates in terms of invasive vs. colonization sites, suggesting that ACME may not contribute to the enhanced virulence in human infection. In addition, a recent study has shown that ACME^+^ and ACME^−^ *S. epidermidis* isolated from neonates induce similar inflammatory responses using a whole-blood model, and ACME is not associated with increased pathogenicity in *S. epidermidis* from neonates [34] further supporting that ACME may not be a key virulence factor for USA300.

Genomic sequencing has revealed that USA300 carries a native *arc* cluster and two *opp* operons on the core chromosome in addition to those on the ACME genomic island in the USA300 genome [4]. Based on the results obtained in the present study, the *arc* cluster and *opp*-3 on ACME might be redundant genes and the virulence of USA300 clone is probably determined by the core genome.

In addition to studying the function of ACME in bacterial pathogenesis, our study provides insight into the movement of ACME as a mobile genetic element in bacteria and the evolution of USA300 strains. Based on the observation of the presence of ACME^−^ USA300 clinical isolates, we have proposed that ACME and/or SCC*mec* may excise spontaneously and be lost during evolution. To test this theory, individual excisions of ACME and ACME-SCC*mec* have been screened using the 2406 WT strain. Colonies with natural excision of SCC*mec* and AMCE-SCC*mec* but not ACME have been isolated. The reason why the colonies with natural excision of ACME were not identified is probably due to the lower frequency of ACME excision compared with that of SCC*mec* and ACME-SCC*mec*, which is illustrated by qRT-PCR assay developed in this study. This may also provide an explanation why the prevalence of ACME^−^ USA300 clinical isolates is still low to date. However, a recent study on evolution of USA300 has observed the increase of SCC*mec*^−^ and ACME^−^ isolates in the USA, supporting the hypothesis proposed here [35]. Furthermore, the qRT-PCR results has also confirmed that the excision is mediated by CcrAB2, because ACME excision was not observed in exSCC*mec*^−^ in which the *ccr*AB2 genes are absent, and the excision of SCC*mec* is not seen in *S. aureus* COL because it has a premature stop codon in the *ccr*B gene [29]. In addition, the excision frequency is not dependent on the size of the excised fragment because SCC*mec* (24 kb) and ACME-SCC*mec* (55 kb) have almost similar excision frequencies, 10^−3.6^ and 10^−3.7^, respectively.

The epidemiology data showed the co-existence of ACME^+^/^−^ clinical isolates in nature, and these isolates have similar genotypic and phenotypic features except for the presence or absence of ACME. There are two possibilities for the emergence of USA300 ACME^−^ isolates. One is that the recent ancestor of USA300 has acquired SCC*mec* first and quickly acquired ACME afterwards, but ACME is lost during evolution. The other possibility is that the ancestor of USA300 has acquired SCC*mec* and ACME at the same time, but ACME is spontaneously lost during evolution. The present study supports the former possibility because the frequency of excision of ACME and SCC*mec* are different, and no linkage between ACME and SCC*mec* IV has been found in other bacteria including *S. epidermidis*. On the other hand, ACME^−^ USA300 clinical isolates have also been found in other geographic areas, such as US and Australia, with low frequency [36,37]. Moreover, USA300 SCC*mec*^−^ and ACME^−^SCC*mec*^−^ clinical isolates have also been identified in these studies. Recently, there was an outbreak of skin infections in college football players that was caused by a USA300 MSSA (SCC*mec*^−^) ACME positive strain [38]. All these reports support the hypothesis that USA300 could spontaneously lose mobile genetic elements including ACME, SCC*mec* or ACME-SCC*mec* in a low frequency to generate new clones.

**Figure 10:**
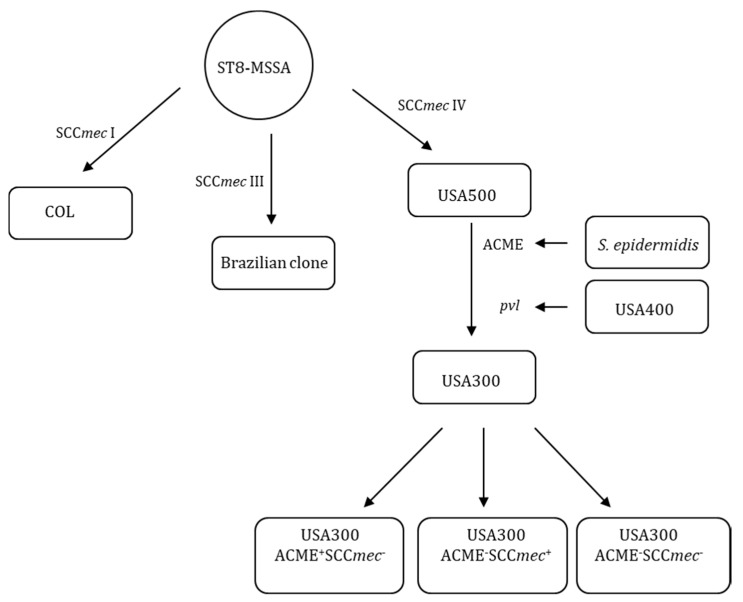
The hypothesized evolution of USA300. ST8-MSSA strains acquired SCC*mec* IV to generate USA500 strains and USA500 strains acquired the PVL and ACME to generate USA300 strains during evolution. The PVL might have originated from USA400 and ACME might have come from *S. epidermidis*. USA300 strains can generate USA300 ACME^+^SCC*mec*^−^, ACME^−^SCC*mec*^+^ and ACME^−^SCC*mec*^−^ strains by losing ACME and/or SCC*mec.* ST8-MSSA strains also acquired SCC*mec* I or III to generate COL or Brazilian strains.

It has been hypothesized that USA300 strains originated from the ancestor strain ST8-MSSA by acquiring SCC*mec* IV, PVL and ACME [39]. The analysis of PVL gene sequence of *S. aureus* collected from different continents suggests that USA300 may have acquired the PVL genes from USA400 [40]. The ACME might have originated from *S. epidermidis* because ACME I.02 (a homolog of ACME in USA300) has been identified in *S. epidermidis* [41,42]. ST8-MSSA also generated other lineages including COL and Brazilian clones by acquiring SCC*mec* I and III [39]. The present study suggests the hypothetical evolution of USA300 can be updated as shown in Figure 10, which USA300 may continue evolving to generate USA300 ACME^+^SCC*mec*^−^, ACME^−^SCC*mec*^+^ and ACME^−^SCC*mec*^−^ strains.

Altogether, this study has demonstrated that although ACME is unique to the genome of CA-MRSA USA300, it may not be a key marker for USA300 virulence and transmission. ACME may be integrated into the USA300 genome by coincidence, and how this happened remains to be answered. Thus, we will probably see an increase of ACME^−^ isolates in the future because these isolates could have better fitness than ACME^+^ isolates if ACME does not have any major role in bacterial virulence and transmission. In addition, the qRT-PCR protocol that has been developed in this study can be used to study the evolution of CA-MRSA since this protocol can monitor the frequency of spontaneous ACME, SCC*mec* and ACME-SCC*mec* excision. However, further studies may be required to determine the function of ACME. We also recognize the limitation of the current study without the experiment with complementation of the mutations due to the technical limitation.

## 5. Conclusions

Our results do not support that the ACME element alone is a significant factor in the transmission and virulence of USA300 strain, and ACME may have been coincidently incorporated into the genome of USA300 strain. In addition, the qRT-PCR protocol that has been developed in this study can monitor the frequency of spontaneous ACME, SCC*mec* and ACME-SCC*mec* excision. Hence, this protocol can be used to study the evolution of CA-MRSA.

## Figures and Tables

**Table 1:** Primers for construction of USA300-2406 Δ*arc*A and ΔACME mutants.

Primers	Sequence (5′ to 3′) ^a^
ACME-LF ^b^	TTTTGTCGACGAATCTATGGCCACTAACTGC
ACME-LR	TTTTGACGTCAGGTGACAAATAAAAGGCTACC
ACME-RF	TTTTATCGATTACACCAGTCATGCTTACAGG
ACME-RR	TTTTCTCGAGCTTTAATCTCTTCGTTTACGACC
*arc*A-LF	TTTTGTCGACTAATTAAAGCGTTGACCG
*arc*A-LR	TTTTGACGTCCTTAATTGGTTTAGTCATAGGC
*arc*A-RF	TTTTATCGATTCACAATTGTTTAGGGAGG
*arc*A-RR	TTTTCTCGAGTAAAGCGCTCTAATACATACC
pGen-F ^c^	AACCCAAGCTTATCGATGAGGGTATTAATAATGAAAGGG
pGen-R	TTCGCGGATCCGACGTCAGGTAATCCTAGAGCTTGCC
*arcA*-ExtF	GGTAAAAAGCACTGAGTGTATATGG
USA300-ExtF	AGCTTAATAAGTTCTACCTTGACC

Restriction sites are underlined: GTCGAC for SalI, GACGTC for AatII, ATCGAT for ClaI, CTCGAG for XhoI. ^b^ Primers-LF and -LR amplify the left flanking region and primers-RF and -RR amplify the right flanking region of target genes. ^c^ Primers pGen-F and pGen-R amplify the gentamicin resistance gene from M92.

**Table 2:** Primers for comparison of gene expression between the wild type and the mutants using RT-PCR.

Primers	Sequence (5′ to 3′)
*gyrB*-F	ATCGACTTCAGAGAGAGGTTTG
*gyrB*-R	CCGTTATCCGTTACTTTAATCCA
*arcA*-F	GCAGCAGAATCTATTACTGAGCC
*arcA*-R	TGCTAACTTTTCTATTGCTTGAGC
*Opp3C-F*	TCTTAGTAAGACTGATTGTCGG
*Opp3C-R*	GAATCACATGTGTTACTGTCG

**Table 3:** Spontaneous excision frequency of ACME and/or SCC*mec* from the chromosome of *S. aureus.*

Strain	Excision/Circularization					
	ACME	SCC*mec*	ACME-SCC*mec*	ACME (cir) ^a^	SCC*mec* (cir)	ACME-SCC*mec* (cir)
USA300-2406	10^−5.5^	10^−3.6^	10^−3.7^	10^−5.7^	10^−5.0^	10^−4.5^
exACME^−^	N/A ^b^	10^−2.7^	N/A	N/A	10^−4.9^	N/A
exSCC*mec*^−^	U/D ^c^	N/A	N/A	U/D	N/A	N/A
COL ^d^	N/A	U/D	N/A	N/A	N/A	N/A
CMRSA2	N/A	10^−6.1^	N/A	N/A	N/A	N/A

^a^ cir indicated ACME and/or SCC*mec* formed circularized DNA after excision from the chromosome; ^b^ N/A, not applicable; ^c^ U/D, undetectable; ^d^ spontaneous excision of SCC*mec* was also tested in other MRSA strains, including COL and CMRSA2, as controls.

## Abbreviations

CA-MRSA	Community-associated methicillin-resistant Staphylococcus aureus
MRSA	Methicillin-resistant Staphylococcus aureus
ACME	Arginine catabolic mobile element
*arc*	Arginine deiminase
*opp*-3	oligopeptide permease
SCC*mec*	Staphylococcal cassette chromosome mec
PFGE	Pulsed field gel electrophoresis
WT	wild type
PCR	Polymerase chain reaction
Δ	deletion
BHI	Brain heart infusion
TSA	Tryptic soy agar
CFU	Colony forming units
CI	Competition index
MEM	Minimum essential medium
FBS	Fetal bovine serum
PBS	Phosphate buffered saline
EDTA	Ethylenediaminetetraacetic acid
LDH	Lactate dehydrogenase
NA	Nalidixic acid
AHS	Alberta Health Services
*spa*	staphylococcal protein A
MLST	Multilocus sequence typing
*agr*	Accessory gene regulator
PVL	Panton-Valentine Leukocidin
qRT-PCR	Quantitative reverse transcription polymerase chain reaction
Ct	cycle threshold
SSTI	Skin and soft tissue infection
MSSA	Methicillin-susceptible Staphylococcus aureus
